# Effects of salvianolate on microcirculatory disturbance in patients with stable coronary heart disease: study protocol for a randomized controlled trial

**DOI:** 10.1186/s13063-021-05099-7

**Published:** 2021-03-08

**Authors:** Zhanlu Li, Yi Luan, Min Wang, Ya Li, Xiaohua Shen, Guosheng Fu, Wenbin Zhang

**Affiliations:** Department of Cardiology, Key Laboratory of Cardiovascular Intervention and Regenerative Medicine of Zhejiang Province, Sir Run Run Shaw Hospital, School of Medicine, Zhejiang University, 3 East Qingchun Road, Hangzhou, Zhejiang 310016 People’s Republic of China

**Keywords:** Salvianolate, Coronary microcirculation

## Abstract

**Background:**

Obstruction of coronary microcirculation can lead to myocardial ischemia and poor prognosis. Salvianolate exerts cardiovascular protection at cellular levels. However, no studies have confirmed the effect of salvianolate on stable coronary heart disease (CHD) with high fractional flow reserve (FFR) and myocardial microcirculatory disturbances.

**Methods/design:**

This study will enroll 78 patients who have stable coronary disease with 50 to 70% stenosis in major coronary arteries and whose FFR > 0.80 and index of microcirculatory resistance (IMR) > 25. Patients will be randomly divided into the salvianolate group or the placebo group. After above evaluations, salvianolate 200 mg will be intravenously dripped immediately for the next 30 min and subsequent 7 days in the salvianolate group, and matching 0.9% normal saline will be arranged in the placebo group. IMR will be reevaluated in immediate phase after first 30 min of salvianolate or placebo treatment. The primary end point will be the IMR change in this phase, and the secondary end points will be the total ischemic burden assessed by the Seattle angina scale, quality of life scale, Holter electrocardiography, and 6-min walk test after 7 days before discharge.

**Discussion:**

This study will firstly clarify the improvement effect of salvianolate on coronary microcirculation and provide an effective treatment method for stable CHD patients with high FFR and myocardial microcirculatory disturbance.

**Trial registration:**

Chinese Clinical Trial Registry ChiCTR1800018772. Registered on 9 October 2018 and updated on 2 March 2020

## Background

Coronary microcirculatory disturbance is one of the most important causes of angina pectoris [1], and it is often difficult to be diagnosed and treated in clinical practice. Index of microcirculatory resistance (IMR) is a novel and relatively simple quantitative assessment method. It can truly reflect the microcirculatory function and is not affected by the epicardial artery stenosis. IMR is recognized as the best indicator to assess the coronary microcirculation damage [2–4]. The simultaneous assessment of fractional flow reserve (FFR) and IMR in patients with critical coronary lesions can clarify the causes of myocardial ischemia in stable coronary heart disease (CHD).

Obstruction of coronary microcirculation can lead to myocardial ischemia [5], significantly affecting the patients’ prognosis [6]. It has been found that angiotensin-converting enzyme inhibitors and sildenafil can improve the microvascular index of patients with microcirculatory disorders [7–9] while therapeutic effects of calcium antagonists and alpha blockers have been highly controversial [10, 11].

Salvianolate is a water-soluble effective active site of traditional Chinese herb salviae miltiorrhizae, and its main component is magnesium lithospermate B. Magnesium lithospermate B can protect the cardiovascular system via anti-oxidation, anti-inflammation, endothelial protection, myocardial protection, anticoagulation, vasodilatation, anti-atherosclerosis, and reducing the proliferation and migration of vascular smooth muscle cells [12, 13]. It can also reduce the incidence of contrast-induced nephropathy after percutaneous coronary intervention and is more effective than normal saline [14]. In addition, it has been confirmed that intravenous administration of salvianolate can improve cardiac microcirculatory perfusion and increase cardiac output in rats [15]. Similarly, continuous intravenous salvianolate 10 mg/kg/day for 7 days can significantly improve myocardial microcirculatory reflow in an ischemia-reperfusion minipig model, as well as reduce oxidative stress and myocardial apoptosis [16]. However, no studies have been conducted to verify the efficacy of salvianolate against coronary microcirculatory disturbance of CHD from the perspective of IMR.

Accordingly, in this study, we try to evaluate the effect of salvianolate intravenous infusion on IMR in stable CHD patients with high FFR and myocardial microcirculatory disturbance, as well as to evaluate the effect of short-term use of salvianolate on total ischemic burden in these patients.

## Methods/design

### Study design

This is a prospective, single-center, randomized, double-blind controlled study. We will enroll 78 stable CHD patients who have a 50 to 70% stenosis in major coronary arteries and whose FFR > 0.80 and IMR > 25. They will be randomly divided into the salvianolate group or the placebo group. The former group will be given intravenous salvianolate (200 mg salvianolate dissolved in 100 ml 0.9% sodium chloride injection, intravenous drip for 30 min) after the first measurement of IMR. The latter group will be intravenously given matching 0.9% normal saline 100 ml. Thirty minutes later, both groups will undergo reevaluation of IMR. For the subsequent 7 days, the salvianolate group will be intravenously infused with salvianolate 200 mg/day and the placebo group will continue to use matching 0.9% normal saline per day. Other medications will be used according to clinical needs. After 7 days, all patients will be assessed by the Seattle angina scale, quality of life scale, Holter electrocardiography, and 6-min walk test.

The trial protocol has been approved by the Institutional Ethics Committee of Sir Run Run Shaw Hospital of Zhejiang University and has been registered in the Chinese Clinical Trial Registry (ChiCTR1800018772). All patients will provide written informed consents before randomization.

### Inclusion and exclusion criteria

Seventy-eight stable CHD patients with 50 to 70% stenosis in major coronary arteries, FFR > 0.80, and IMR > 25 at Sir Run Run Shaw Hospital of Zhejiang University will be enrolled in this trial. Detailed inclusion and exclusion criteria are shown in Table 1. The attending physician of the patient is responsible for inclusion and exclusion assessment.

**Table 1:** Inclusion and exclusion criteria

Inclusion criteria	Exclusion criteria
• Angina pectoris • CAG showed 50 to 70% stenosis in major coronary arteries; FFR > 0.8, IMR > 25; • Patients received no salvianolate treatment previously.	• Patients with left main disease; • Patients with severe cardiac insufficiency (New York Heart Association grade III or higher), severe renal insufficiency (eGFR < 60 ml/min), liver dysfunction (ALT > 3 × URL), and asthma history; • Severe cerebrovascular disease; infection, hematopoietic dysfunction, tumor or mental illness; • Combined malignant arrhythmia or third degree atrioventricular block; • Currently taking other Chinese herbs for the treatment of angina; • Pregnant or breastfeeding women; • Currently participating in other clinical trials; • Allergic to known ingredients of salvianolate; and • The investigator considers it unsuitable for inclusion in other circumstances, or the patient or family members do not agree with the study’s treatment plan.

*CAG*, coronary angiography; *FFR*, fractional flow reserve; *IMR*, index of microcirculatory resistance; *eGFR*, estimated glomerulus filtration rate; *ALT*, alanine aminotransferase; *URL*, normal reference level

### Exclusion criteria during the follow-up periods

Patients who should not be enrolled but have been enrolled should be excluded. Patients who have received at least one dose of salvianolate or matching placebo but fail to complete the trial because of poor compliance or voluntary withdrawal should not be removed. Every withdrawal will be recorded in the case report forms (CRFs), and a visit will be arranged if possible. Although their data cannot be used for efficacy analysis, they can be used for safety analysis. The trial could be terminated in advance by investigators if there are any safety problems. Once it is terminated, all CRFs should be kept for future reference.

### Implementation and study procedures

**Fig. 1:**
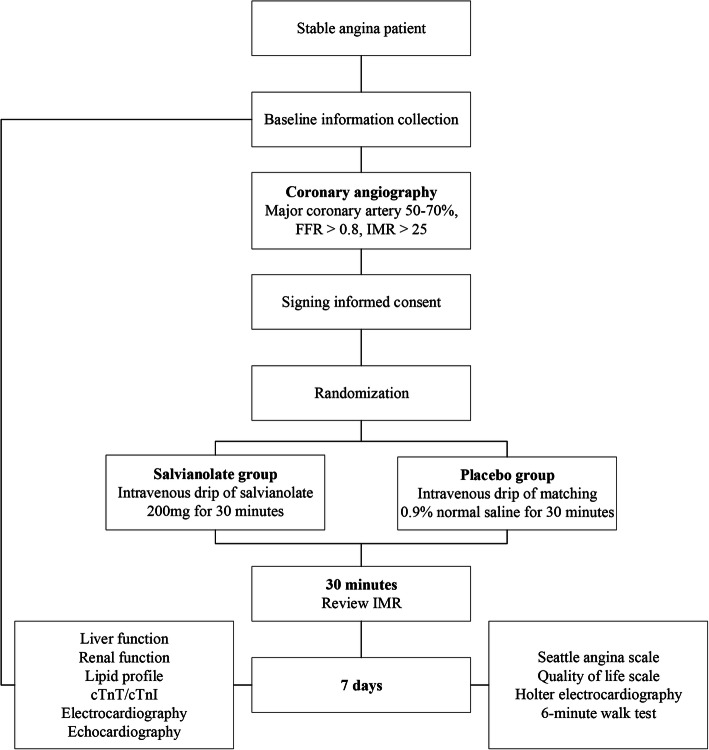
Flow chart. FFR, fractional flow reserve; IMR, index of microcirculatory resistance; cTnT/cTnI, cardiac troponin T/I

In this study, intravenous drip of salvianolate will be performed during coronary angiography and the subsequent 7 days and its effects on microcirculatory disturbance will be assessed via the IMR change and total ischemic burden. The study protocol is shown in Fig. 1 and Table 2, which is divided into three phases, including screening, immediate phase, and in-hospital follow-up.

**Table 2:** The study plan

	Screening	Immediate phase (intravenous drip of salvianolate for 30 min)	In-hospital follow-up
	Day 1	Day 1	Day 7
Inclusion/exclusion criteria	X		
Informed consent	X		
Randomization	X		
Demographic information	X		
Medical history	X		
Vital signs	X	X	X
Physical examination	X		X
Concomitant medications	X		X
CBC	X		X
Scr, BUN, UA	X		X
K, Na, Cl	X		X
ALT, AST, BIL	X		X
Fasting glucose/lipids	X		X
cTnT/cTnI	X		X
Electrocardiography	X		X
Echocardiography	X		X
**Medication**			
Salvianolate injection		X	X
**End points**			
Seattle angina scale	X		X
Quality of life scale	X		X
Holter electrocardiography	X		X
6-min walk test	X		X
IMR	X	X	

*CBC*, complete blood count; *Scr*, serum creatinine; *BUN*, blood urea nitrogen; *UA*, uric acid; *K*, potassium; *Na*, sodium; *Cl*, chloride; *ALT*, alanine aminotransferase; *AST*, aspartate aminotransferase; *BIL*, bilirubin; *cTnT/cTnI*, cardiac troponin T/I; *IMR*, index of microcirculatory resistance

In the screening stage, the investigator conducts a baseline assessment and gets the informed consent and randomization. Coronary angiography, FFR, and IMR will be used to determine whether the inclusion and exclusion criteria are met, and then informed consent should be signed by the patient or the authorized person. The web-based randomization assigned those eligible patients 1:1 ratio to either the salvianolate or the placebo group. The secretary of the catheterization room accesses the website via account password and is responsible for randomization. Block randomization stratified by sex will be implemented and it is blind to those who enroll participants or assign interventions. In the immediate phase, the patients will receive an IMR test again after 30 min of intravenous infusion and the research nurses will record the IMR value. In the in-hospital follow-up period, complete blood count (CBC), serum electrolytes (potassium (K), sodium (Na), chloride (Cl)), liver function (alanine aminotransferase (ALT), aspartate aminotransferase (AST), bilirubin (BIL)), renal function (serum creatinine (Scr), blood urea nitrogen (BUN), uric acid (UA)), fasting glucose, lipid profile, cardiac troponin T/I (cTnT/cTnI), echocardiography, Seattle angina scale, quality of life scale, Holter electrocardiography, and 6-min walk test will be collected after continuous intravenous salvianolate 200 mg/day for 7 days.

All serious adverse events must be reported to the sponsor immediately or within 24 h in CRFs. The attending physicians are responsible for data entry. The data monitoring committee is responsible for data security and accuracy. Committee members (Zhimin Xue, Juhong Zhang, Xiaoting Li, and Fen Xu) have access to the final dataset and periodically range check for data values.

### End points

The primary end point will be the IMR change in the immediate phase during the coronary angiography procedure, and the secondary end points will be the total ischemic burden assessed by the Seattle angina scale, quality of life scale, Holter electrocardiography, and 6-min walk test after 7 days before discharge. The safety end point will be the abnormal blood tests and self-reported adverse events.

### Trial committees

Wenbin Zhang guides the research team and provides overall supervision for the trial. The data monitoring committee (Zhimin Xue, Juhong Zhang, Xiaoting Li, and Fen Xu) is responsible for reviewing and monitoring data safety. They need to make sure that all CRFs are filled out correctly and consistent with the original data and that all adverse events have been recorded and all serious adverse events have been reported in accordance with the relevant procedures. Investigators have the right to reveal a participant’s allocated intervention or terminate the study in advance after discussion with Wenbin Zhang in the event of safety issues during the study as well as notify the ethics committee.

### Amendments

The protocol should be strictly implemented as it has been approved by the ethics committee. If it is necessary to add or revise the study protocol after the start of the study, the investigator should submit it to the ethics committee again and obtain approval before implementation. The modification of the protocol will be recorded in detail. The trial was registered on 9 October 2018 and updated on 2 March 2020 (URL: http://www.chictr.org.cn).

### Auditing and dissemination policy

To ensure the trial is carried out in accordance with the standard operating procedures and corresponding regulatory requirements, an independent audit, i.e., systematic and independent review of research-related activities and documents, can take place at the research center at any time during or after the end of the study. The trial results will be published in the form of a paper or presentation, and the name of the patients and other personal information will not be disclosed (excluding the date of birth/age and gender).

## Statistical considerations

### Sample size

We use the URL (https://www.cnstat.org/samplesize/5/) to generate the calculated sample size. According to our pre-experimental results, we assume *α* = 0.05, 1-*β* = 0.80, Mean_*t*_ = 20, Mean_*c*_ = 40, *σ* = *S*_*t*_ = *S*_*c*_ = 20, *N*_t_:*N*_c_ = 1:1, and suppose that a 20% IMR reduction (Δ = − 8) is considered of clinically meaningful difference. We get approximately 36 (between 35 and 36) needed patients in each group. Considering the loss rate of 10%, the total sample size will be 78 cases with 39 cases in each group. The equation being used to estimate the sample size is as follows:

$$ n=\frac{2{\left({Z}_{\alpha }+{Z}_{\beta}\right)}^2{\sigma}^2}{{\left({\mathrm{Mean}}_t-{\mathrm{Mean}}_c-\Delta \right)}^2}. $$

### Analysis

All data will be presented as percentages, mean ± standard deviation (SD), or median (quartile range). As the population variance *σ*^2^ is unknown, the confidence interval of the population mean *μ* is calculated by the formula $$ \overline{X}\pm \frac{S}{\sqrt{n}}{t}_{\alpha}\left(n-1\right) $$. Symbol $$ \overline{X} $$, *s*, and *n* are the sample mean, SD, and sample size, respectively, while *α* represents the significant level. The confidence interval of the difference between means will also be calculated by $$ \overline{X}\pm {S}_{{\overline{X}}_1-{\overline{X}}_2}{t}_{\raisebox{1ex}{$\alpha $}\!\left/ \!\raisebox{-1ex}{$2$}\right.}\left({n}_1+{n}_2-2\right) $$, in which $$ {S}_{{\overline{X}}_1-{\overline{X}}_2} $$ is the standard error of the difference of means. The adjusted Wald confidence intervals are used to estimate confidence intervals for proportions and the difference between the two population proportions [17].

IMR changes between the two groups will be compared by independent *t*-test or Mann-Whitney *U* test, while IMR changes before and after the use of salvianolate in the same group will be compared by paired *t*-test or Wilcoxon test. Analysis of covariance will be used if the baseline and follow-up IMRs are not highly correlated (*r* ≤ 0.8) [18, 19]. If the variables are not normally distributed, they will be transformed into normal distribution by logarithm. Different hypothesis testing, including *t*-test or nonparametric tests for continuous variables as well as *χ*^2^ test for categorical variables, will be used for the secondary end points assessed by the Seattle angina scale, quality of life scale, Holter electrocardiography, and 6-min walk test. The safety end point in our trial will be the abnormal blood tests including the abnormal liver function and self-reported adverse events such as dizziness and headache. The rate of abnormal liver function and other adverse events between the salvianolate group and the placebo group will be compared by *χ*^2^ test.

In our trial, stable CHD patients with myocardial microcirculatory disturbances form the population of interest, and participants with 50 to 70% stenosis in major coronary arteries, FFR > 0.80, and IMR > 25 form a sample. The end point of interest will be the IMR change in the immediate phase and the total ischemic burden assessed by the Seattle angina scale, quality of life scale, Holter electrocardiography, and 6-min walk test in the follow-up period.

Premature discontinuation of salvianolate, loss to follow-up, and death are possible intercurrent events. As we use treatment policy strategy in our study, we will take a per-protocol analysis, as well as intention-to-treat (ITT) analysis for statistical analysis. Per-protocol analysis includes participants who complete the assigned therapy according to the trial protocol while ITT analysis includes all randomly assigned participants according to the treatment group to which they were originally assigned, regardless of the occurrence of intercurrent events. The ITT analysis will indicate how salvianolate will work in a more real-life setting. If there is evidence that the difference in the treatment depends on certain patient characteristics identified in the baseline assessment, a subgroup analysis will be performed.

The missing data before the intercurrent events will be filled with the measured values of “similar participants.” The similar patients should have the same sex and smoking history (smoker or nonsmoker). Missing values will be imputed by expectation-maximization algorithm through age, systolic blood pressure, diastolic blood pressure, fasting blood glucose, and IMR. Multiple imputation will be used in our study. *P*-value < 0.05 will be considered to indicate statistical significance. All statistical analyses will be performed using the Statistic Package for Social Science (SPSS) software package, version 18.0 (SPSS Inc., Chicago, IL, USA). The results will be published by the chief investigator and major contributors.

## Discussion

Traditional methods for improving angina include drug therapy, interventional therapy, and coronary artery bypass grafting. However, up to 49% of patients with angina have no significant coronary artery stenosis [20, 21]. Among them, 50 to 65% of patients have impaired coronary vasodilatation, resulting in coronary microcirculatory disturbances [1]. The European Society of Cardiology 2013 guideline for stable CHD clearly incorporates coronary microcirculatory disorder into the field of stable CHD and proposes the concept of CHD patients with ischemic symptoms but no evidence of obstruction [22]. Their risk of long-term cardiovascular events (mortality, hospitalization for acute coronary syndrome and stroke) is comparable to obstructive coronary disease [23, 24]. The tiny blood vessels of the coronary circulation mainly nourish the myocardium and cardiac autonomic nerves and play a crucial role in the regulation of myocardial metabolism [25]. Even in the absence of obstruction of the epicardial coronary artery, microvascular contraction and relaxation can also regulate coronary blood flow and myocardial perfusion, directly affecting cardiac function.

Risk factors for CHD such as smoking, old age, hyperlipidemia, diabetes, and mental stimulation can damage small coronary endothelium cells and affect coronary microcirculation [26]. Endothelial and smooth muscle dysfunction, sympathetic nerve dysfunction, microvascular atherosclerosis, and inflammation are closely related to microcirculation disorders [27]. The treatment of coronary microcirculation disorders can only rely on drugs, but currently, there is no clear and effective treatment of drugs. Salviae miltiorrhizae is a traditional Chinese herb and its effective ingredient salvianolate can protect the cardiovascular system at cellular levels [12]. It can inhibit the production of reactive oxygen species and increase the antioxidant capacity of cardiomyocytes [28], as well as improve microvascular reflow by inhibiting oxidative stress and apoptosis [16]. Previous study has demonstrated that five salviae miltiorrhizae-based preparations, including salvianolate injection, were effective in the treatment of stable angina pectoris with clinical improvement rate of 72.4 to 91.6% and electrocardiography improvement rate of 54.5 to 71.6% [29]. However, the effect of salvianolate intravenous infusion on the myocardial IMR in patients with high FFR and myocardial microcirculatory disturbance remains unclear. Accordingly, we focus on this issue and further explore the improvement effect of salvianolate on angina symptoms and myocardial ischemia.

Nonetheless, there are still limitations in this study. First, this study is a single-center participation study. Second, salvianolate will be compared with placebo only in this study, and there are no other comparative groups for effective drugs for coronary microcirculatory disturbances, such as angiotensin-converting enzyme inhibitors. Other non-inferiority and superiority trials may be needed. Although the study could only be designed based on the aforementioned limitations, we can still evaluate the effect of intravenous salvianolate infusion on IMR in stable CHD patients with high FFR and myocardial microcirculatory disturbance and test the improvement of the clinical symptoms of CHD patients with stable microcirculatory disturbance.

In conclusion, this study will clarify the effect of salvianolate on coronary microcirculation and provide an effective treatment for patients with stable CHD with high FFR and myocardial microcirculatory disturbances.

### Trial status

Chinese Clinical Trial Registry ChiCTR1800018772, version 2. Registered on 9 October 2018 and last updated on 2 March 2020. Participant recruitment began in January 2020 and is expected to continue until 2022. Randomization of the participants was performed on the same day.

## Data Availability

Not applicable.

## Abbreviations

IMR: Index of microcirculatory resistance
FFR: Fractional flow reserve
CHD: Coronary heart disease
CRFs: Case report forms
CBC: Complete blood count
K: Potassium
Na: Sodium
Cl: Chloride
ALT: Alanine aminotransferase
AST: Aspartate aminotransferase
BIL: Bilirubin
Scr: Serum creatinine
BUN: Blood urea nitrogen
UA: Uric acid
cTnT/cTnI: Cardiac troponin T/I
SD: Standard deviation
ITT: Intention-to-treat
SPSS: Statistic Package for Social Science
